# An assessment of factors contributing to treatment adherence and knowledge of TB transmission among patients on TB treatment

**DOI:** 10.1186/1471-2458-4-68

**Published:** 2004-12-29

**Authors:** Frederick AD Kaona, Mary Tuba, Seter Siziya, Lenganji Sikaona

**Affiliations:** 1 Mwengu Social and Health Research Centre, 12 Kafupi Road, Plot number 1430/130, Northrise P.O Box 73693, Ndola, Zambia; 2 University of Zambia School of Medicine, Department of Community Medicine, P.O Box 50110, Lusaka, Zambia; 3 Ministry of Agriculture Food and Fisheries Headquarters, Box RW 50291, Lusaka, Zambia

## Abstract

**Background:**

The treatment guidelines for tuberculosis treatment under Directly Observed Treatment, Short-course (DOTS) have been a common strategy for TB treatment in Zambia. The study was carried out in Ndola, Zambia, to investigate factors contributing to treatment non-adherence and knowledge of TB transmission among patients on TB treatment, in order to design a community-based intervention, that would promote compliance.

**Methods:**

A household-based survey was conducted in six randomly selected catchment areas of Ndola, where 400 out of 736 patients receiving TB treatment within the six months period, were recruited through the District's Health Management Board (DHMB) clinics. All patients were interviewed using a pre-tested structured questionnaire, consisting of i. Socio-demographic characteristics ii. Socio-economic factors iii. Knowledge about TB transmission and prevention iv. Patterns in health seeking behaviour and v. TB treatment practices at household level.

**Results:**

Most male TB patient respondents tended to be older and more educated than the female TB patient respondents. Overall, 29.8% of the patients stopped taking their medication. There were 39.1% of the females and 33.9% of the males, who reported that TB patients stopped taking their medication within the first 2 months of commencing treatment. Age, marital status and educational levels were not significantly associated with compliance. The major factors leading to non-compliance included patients beginning to feel better (45.1% and 38.6%), lack of knowledge on the benefits of completing a course (25.7%), running out of drugs at home (25.4%) and TB drugs too strong (20.1% and 20.2%). There was a significant difference [OR = 1.66, 95% CI 1.23, 2.26] in TB knowledge, with more males than females reporting sharing of cups as a means for TB transmission, after adjusting for age, marital status and educational levels. Significantly [p = 0.016] more patients who had resided in the study for less than two years (59%) were more likely to report mother to child transmission of TB, compared to 41.2% of those who had been in the area for more than 2 years.

**Conclusion:**

This study established that 29.8% of TB patients failed to comply with TB drug taking regimen once they started feeling better.

## Background

Tuberculosis is one of the major public health risk factors of high mortality among patients in Zambia. The prevalence of tuberculosis among adults has more than doubled since the onset of AIDS epidemic in Zambia [1], with most of the cases often related to HIV/AIDS conditions. Because of the advent of AIDS epidemic in the country, tuberculosis cases have increased to over 500/100,000 population [2]. Due to AIDS, it has been projected that TB cases would reach 38,000 by the year 2004 in Zambia [3]. A multisite study conducted in Ndola in 1997 [4] revealed that HIV prevalence was as high as 42%, and in a sub-sample of women attending antenatal clinics (ANC), HIV prevalence rate was 28.4% [5]. Data from Ndola and other Zambian communities revealed that the number of TB cases had risen five-fold since 1996 [6]. Because of the communities' health seeking behaviour and their perceived causes of TB among patients, those suffering from TB would transmit the disease to others and merging drug-resistance strains would make it even more difficult to limit the spread of the infection [1,3].

Although tuberculosis treatment guidelines had been circulated for the management of the disease at health facility down to household level, DOTS was the only best means of increasing compliance among TB drug-taking patients in Zambia [1,7]. As early as 1980s, the African countries had embraced and implemented the WHO recommended DOTS strategy, which was meant to standardize methods for case detection, case management and monitoring [8]. DOTS as a strategy, entails that medication is taken in while the care provider is observing the patient swallowing the drug [9]. Diagnosing TB patients at a health facility and sending them to continue taking their treatment at home, poses serious challenges for other members of the households who assist the patients with treatment.

Work carried out in Haiti [7], Zambia [10] and most other rural communities [11] present the challenges facing compliance with TB treatment. Tuberculosis like HIV/AIDS is often associated with stigmatization and thus may create resistance among patients to treatment. A study carried out in Nigeria [12], raised an important point of delays in care seeking behaviour due to stigma experienced by TB patients. Studies [13] have shown that stigmatization creates a lot of self-denial among those with diseases like TB and Sexually Transmitted Infections (STIs); hence most of them fail to comply with the treatment regime.

Issues of diverse nature to caring of patients at home are rampant in communities that have low literacy levels [13,14]. The possibility of being exposed to tuberculosis by merely associating with patients may create some resentment by those in the household to providing proper care of the patient and encourage non-compliance [15,16]. In their studies [4,15,18], researchers found that fear of catching the disease was a factor in household members' negative reactions to care of the TB patient. The high incidence of chronic TB-related problems among the patients increases the probability that care of TB patients becomes costly at home, in terms of food provision and other essentials [18,19]. While TB treatment lasts between 6 and 8 months, the effective treatment of multidrug resistant may be more pronounced in low-income communities and can even be more complex if not properly supervised.

Discrimination on the basis of disease at health facilities sometimes exacerbates problems with compliance with tuberculosis drug taking behaviour [19]. Unless there is privacy during drug collection schedules, patients may resist going to collect their drug supplies at the clinic because of discriminatory behavior by health care providers. Some studies that have been carried out in Africa and other parts of the world [15,19] found that health care providers discriminated against AIDS patients. The type of language used at both the health facility and the homes has a strong bearing on the reactions patients may have and their compliance with treatment. Where TB is perceived to be AIDS, a disease that is frightening and a lurking cause of premature death, those associated with it may sometimes withdraw themselves from the rest of the community because, they believe they have reached the end of their lives.

More often than not, when patients are diagnosed as having TB, communities immediately construct them as social and sexual misfits in the society, which is often followed by exclusion from social interactions and relationships [20]. In homes where care is given, a patient may experience feelings intense loneliness and abandonment. A study among the Nigerian medical students [21] revealed that the majority of the students considered AID a divine punishment for sexual excess. In order to try and improve community participation, organisations such as WHO have developed strategies to enhance TB/HIV/AIDS control [22]. This paper presents factors contributing to treatment adherence and knowledge of TB transmission among patients on TB treatment.

## Methods

### Study design and population

A cross sectional study was conducted among tuberculosis patients who were getting their drugs from the clinics operational in Ndola, Zambia by the Ndola District Health Management Board. In this study, compliance refers to patients who took their TB drugs daily for 8 months. On the other hand, any patient who stopped taking TB drugs during the treatment period was regarded as non-compliant patient. This study took into account an analytical approach of comparing two groups (the compliant against the non-compliant group). The study was limited to these patients because of the research focus on compliance of patients over time to prescribed TB drug regimens identified from medical records. In addition to medical records, TB patients were also directly asked if they had ever stopped taking the drugs since starting the treatment.

### Sample

Using the medical records from the six sampled Ndola District Health Management Board Clinics, a list of all TB patients seeking treatment within the six months period was drawn. A total of 736 patients were listed from the six study sites. Using this figure, and a prevalence of 50% +_5% at 95% confidence level, the minimum sample size was determined to be 248. Although 400 TB patients were recruited in the study, 18 were excluded from the analysis because only demographic data was obtained. Reasons for incompleting questionnaires included some patients becoming restless and rushed to the hospital, while others were too sick to continue with the interview and had died by the re-appointment date.

TB patients were classified as follows: those who never attended the TB-clinic after diagnosis, those who attended 1–2 times after diagnosis and commencement on treatment, and those who attended regularly (i.e. 3 or more times) after diagnosis and continuation of treatment. Patients who attended the clinic regularly and continued taking the treatment were classified as controls, and the rest of the patients were classified as cases.

Out of 18 clinics that were run by the District Health Management Board in Ndola, six were randomly selected from the list of clinics so as to enable the team obtain a representative sample of the clinics in the district. In the sampled clinic catchment areas, a household-based survey was conducted and all patients that met the criteria were invited to take part in the study. In all, 114 non-compliant and 268 treatment-compliant TB patients participated in the study.

### Ethical considerations

TB is strongly associated with HIV/AIDS because of the perceived mode of transmission, which stigmatises and discriminates patients. These are common in most African communities [24] and Zambia in particular [25]. TB like HIV/AIDS becomes difficult to discuss in public. Looking at the sensitive nature of the study, all patients were assured of confidentiality and anonymity. Information on patients, residential addresses, and health facilities to which patients were affiliated were collected for the purpose of follow-up. Respondents were informed that this information would not be made available to persons outside the study team. Respondents were further assured that no person-identifiers would be used for publication.

### Data collection, management and analysis

Those who had agreed to participate were interviewed using a pre-tested structured survey instrument. Data collected were individual socio-demographic characteristics and economic characteristics, knowledge on TB transmission, prevention and treatment practices, patterns in health seeking behaviour, compliance and non-compliance to TB drug taking, and perceived community support during the drug taking period.

The data was entered in EPI6. It was edited using consistency and range checks after data entry. However, data analysis was done in the statistical package, SPSS. The Chi-squared Yates corrected test for 2 by 2 tables and Pearson's uncorrected Chi-squared test for higher contingency tables were used to determine associations between qualitative factors. A multivariate logistic regression model was used to adjust for differences in the distributions of age, marital status and education between males and females in the relationships between knowledge items and gender. The cut off point for statistical significance was set at the 5% level.

## Results

The demographic characteristics of household-based patient population that participated in the survey are presented in Table 1. Information on gender was not recorded for two respondents. Most of the male patient respondents (44.0%) were in the age group 30–39 years, while the majority of female patient respondents (38.2%) were of age 20–29 years (p = 0.001). Among women, TB patients were 2.04 times more likely to be in the age group 10–19 years, compared to males in the same age group. Significantly more females than males were either divorced/separated or widowed (p < 0.001). In terms of educational attainment, males tended to be more educated than females (p < 0.001).

**Table 1:** Socio-demographic characteristics of Patients by Gender

	Total		Male		Female	
Demographic characteristics	n	%	N	%	n	%
AGE [p = 0.001] (years)	Total = 379		Total = 193		Total = 186	
< 10	22	5.8	10	5.2	12	6.5
10–19	20	5.3	5	2.5	15	8.1
20–29	116	30.6	45	23.2	71	38.2
30–39	145	38.3	85	44.0	60	32.3
40–49	47	12.4	31	16.1	16	8.6
50+	29	7.7	17	8.8	12	6.5

MARITAL STATUS [p < 0.001]	Total = 379		Total = 194		Total = 185	
Married	168	44.3	108	55.7	60	32.4
Single	77	20.3	43	22.2	34	18.4
Divorce/Separated	69	18.2	25	12.9	44	23.8
Widowed	65	17.2	18	9.3	47	25.4

EDUCATION [p < 0.001] (Years in school)	Total = 342		Total = 178		Total = 164	
0–4	52	15.2	21	11.8	31	18.9
5–7	146	42.7	60	33.7	86	52.4
8–9	73	21.3	42	23.6	31	18.9
10+	71	20.8	55	30.9	16	9.8

Table 2 shows only one significant difference in knowledge of TB transmission by gender. Significantly more males than females stated that sharing of cups as a means of transmitting TB (27.3% of males and 17.2% of females; p = 0.025). Males were still more likely to hold this belief even after adjusting for age, marital status and education.

**Table 2:** Knowledge on TB Transmission by Gender

	Male		Female			
	Total = 194		Total = 186			
Sources of transmission	n	%	n	%	p value	OR (95% CI)*
Through sexual intercourse	24	12.4	25	13.4	0.875	0.91 (0.62, 1.31)
From mother to child	71	36.6	77	41.4	0.393	0.93 (0.73, 1.19)
Sleeping in same room with TB patient	10	5.2	7	3.8	0.684	1.03 (0.58, 1.82)
Sharing cups	53	27.3	32	17.2	0.025	1.66 (1.23, 2.26)
Patient coughing directly at others	15	7.7	21	11.3	0.313	0.65 (0.42, 1.02)

* OR (95%CI) [Odds Ratio and 95% confidence interval] adjusted for age, marital status and education

No significant differences by duration of residence in the community were observed in knowledge of TB transmission through sexual intercourse, sleeping in the same room with TB patient and patient coughing directly at others (Table 3). Significantly (p = 0.016) more patients with duration of stay of less than two years in the community (59.3%) reported that TB could be transmitted from mother to child compared to 41.2% of patients who had stayed for two or more years in the compounds. Furthermore, significantly (p = 0.019) more patients who had stayed for at least two years in the compounds (27.4%) stated that TB could be transmitted through sharing of cups compared to 11.9% of patients who had stayed in the compounds for less than two years.

**Table 3:** Associations between knowledge of TB transmission and duration of stay in the compound

	Duration of stay in compound				
		
Knowledge of TB transmission	<2 years		2+ years		p value
	Total = 59		Total = 277		
	n	%	n	%	
Through sexual intercourse	9	15.3	40	14.4	0.966
From mother to child	35	59.3	114	41.2	0.016
Sleeping in the same room with TB patient	3	5.1	13	4.7	1.000
Sharing cups	7	11.9	76	27.4	0.019
Patient coughing directly at others	5	8.5	30	10.8	0.762

A total of 114 (29.8%) out of 382 patients stopped taking TB drugs at some point during the treatment regimen. Analyses for the factors associated with TB drug compliance revealed that sex, education, marital status, sharing a room with any one else, relationship with head of household, anyone suffered from TB in the house, close relative or friend that has suffered from TB, number of times suffered from TB, and use of traditional healers for treatment of TB were not significantly associated with compliance.

The common reason given for stopping treatment by both the compliant and the non-compliant patients were that they could not continue with the medication when they started feeling well (45.1% and 38.6%), respectively. Meanwhile, other reasons given by complaint patients were lack of knowledge on the benefits of completing TB course (25.7%), TB drugs too strong (20.1%) and lack of food in the home (11.4%). Similarly, the non-compliant patients mentioned running out of drugs at home (25.4%), TB drugs too strong (20.2%) and loss of hope to live (16.7%) as reasons for stopping.

Common reasons given for stopping treatment for male patients were that they ran out of drugs at home (29.1%) or had no food (23.6%). Meanwhile, most females reported that forgetting to take the medicine (32.0%), reaction to drugs (20.0%), and running out of drugs at home (20.0%) as the reasons for stopping taking drugs (Table 4).

**Table 4:** Perceived Reasons Given by Compliant and Non-compliant Patients Leading to Stoppage of TB Drug Taking

Reason	Compliant		Non-compliant	
	n = 268	%	n = 114	%
Once they start feeling better	121	45.1	44	38.6
Lack of knowledge on the benefits of completing a course	69	25.7	14	12.3
Running out of drugs at home	15	5.6	29	25.4
TB drugs too strong to continue	54	20.1	23	20.2
Lack of food	40	14.9	13	11.4
Loss of hope to live	30	11.2	19	16.7
Lack of drugs at the clinic	5	1.9	5	4.4
Denial of suffering from TB	3	1.1	6	5.3
Doctors advice	0	0.0	2	2.0

Figures cannot add up because the question allowed for multiple responses

Results from the qualitative study reveal that 10 out of 17 non-compliant patients stressed running out of drugs as a reason for stopping. A young man who was a widower aged 32 years reported:

Okay, the drugs got finished but like I am not feeling well, that is why I don't go to collect, because here, there are no children you send. My nephew also has started what? The one who used to get it for me [referring to the nephew who had started work]

In another in-depth interview with a single non-compliant female patient aged 31 years, emphasised on running out of drugs at home and reported thus:

That one we drink two tablets, I..., we went on Monday, they said the Doctor was not there [referring to the clinical officer or nurse responsible for running the TB clinic]... he comes on Wednesday, so I have missed also.

The key determinant for stopping medication in both qualitative and quantitative was running out of drugs.

**Figure 1:**
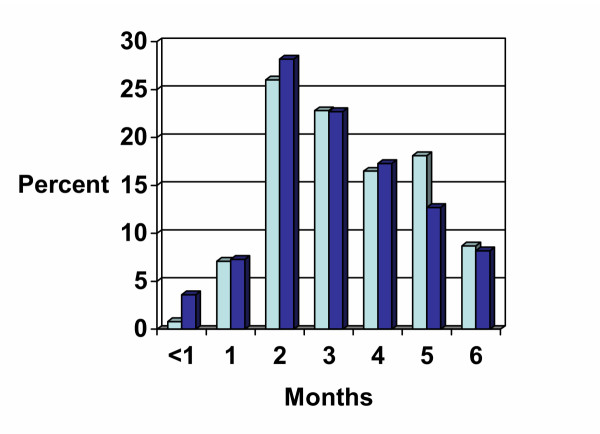
Months since starting treatment at which most TB patients stopped taking drugs  Male  Female

No significant gender differences existed at the stage at which most TB patients stopped taking drugs (p = 0.743). About a third of the respondents (33.9% males and 39.1% females) indicated that they stopped taking drugs within the first two months of starting treatment. However, by the 5^th ^month of consistently taking medication, more men tended to stop taking their medicine (Figure 1). Most of the patients received the drugs from health facilities which were normally very close to their residences. Easy access to drugs created one more problem; where individuals failed to follow the drug schedules; they moved to the next health facility in the other neighbourhood and started their medication. In the new place, they gave false addresses as well as different names from those they were known in their neighbourhood. These are interesting findings that highlight why compliance continues to be difficult in most of the communities.

## Discussion

The Ndola study showed that 29.8% of all TB patients on treatment did not comply. The majority of patients who participated in the study were between 20 and 39 years. This population is in agreement with other studies that had been conducted in Ndola [4,5,24]. It was striking to note that females in the age group 10–19 years were more likely to suffer from TB than their male counterparts in the same age category. Interestingly, this result is consistent with findings from some HIV/AIDS studies conducted in Zambia, which showed higher prevalence of HIV among females in this age group [1,3]. In terms of marital status, more males were identified as divorced/separated or widowed. The educational profile in this study was identical to that of the general population in Zambia [17,21,27]. Most patients had attended primary level of education, with more male patients in the majority. However, there was no relationship between the individuals' level of education and TB infection rates. TB is a serious public health issue that needs continued attention.

It has been demonstrated in other studies [22,26] that the TB epidemic may have changed the population's economic needs, resulting in forcing more young men and women to be exposed to situations that place them at higher risk for TB transmission, for instance, sharing overcrowded rooms even when the situation does exacerbate the risk of TB transmission.

### Knowledge about TB transmission

No relationship has been found between marital status of an individual and the knowledge of TB. Lack of knowledge makes TB a serious public health problem, which needed an urgent attention. TB patients in Ndola could have continued sharing a room with an infected person, because of lack of accomodation, high rental costs and inadequate knowledge on TB transmission. Over 70% of TB patients easily mentioned the symptoms which included sweating at night, loss of weight, loss of appetite and prolonged coughing, which at times was accompanied by sign of blood in the sputum. Knowledge about the symptoms of the disease did not vary according to levels of education or age.

Knowledge modes of transmission and methods of prevention required revisiting, as community members seemed to have had knowledge that did not relate the disease to the environment (that TB could be transmitted through the air). They believed in physical contacts with objects, for instance, sharing of cups, having sexual intercourse with TB patient and from mother to child. This result is consistent with findings from a study in Botswana [19], which reported that TB was a result of pollution from breaking taboos.

It is not surprising therefore, to find no gender differences in terms of TB knowledge transmission among the age groups. The low knowledge in preventive measures in this group could be explained through our study sample itself. Although these age groups were of patients who could have received the information from the clinic during the time they were collecting drugs and their food supplies, which were often provided to patients, prevention did not seem to have been stressed by health care providers. The other minor reason could be that our questions were prompted; this could also explain why 13.3% reported that to prevent TB one should always keep to one partner. The care seeking behaviour remained the same as in other studies [25,26], where the majority of patients went to specialised health facilities once they were sick.

### Duration of residence

The patients' duration of residence in the city of Ndola compared very well with another study [26], which found the median period for most women to be 10 years. It has been shown in this study that duration of residence was associated with the knowledge of tuberculosis transmission.

However, the study had identified some misconceptions on TB transmission, about a third of the respondents reported having got the diseases through sexual intercourse. This study suggested a relationship between community constructions of TB and HIV/AIDS campaigns. Patients could have related TB with HIV and selected sexual intercourse as a source of transmission for the disease, and therefore considered condom use as a means for preventing it. One explanation for this misconception could be attributed to the overstressing that TB is an opportunistic disease for HIV/AIDS. The study in Haiti [9], clearly showed that patients who were diagnosed as TB patients ended up being HIV positive. This misconception was critical in changing people's behaviour and therefore required an urgent intervention.

### Non-compliance

Non-compliance to TB drug taking is reasonably lower than we originally thought. There were 29.8% of the patients who stopped taking their medicine at two months after commencing treatment. The defaulting progressed in the second month (16.8%), and then stabilized thereafter. Factors associated with defaulting in the current study, included patients beginning to feel better, lack of knowledge on the benefits of completing a course, running out of drugs at home and TB drugs being too strong to continue. However, the major and stricking determinant of non-complaince was the patient beginning to feel better.

While lack of food and other provisions were identified in other studies [17], as factors associated with defaulting, in this study lack of food did not come out as an issue. This could be due to the fact that the DHMB through the health facilities distributes food portions (herps, mealie meal, beans and cooking oil) as complimentary food availability at household level for TB patients. In the study at Lusaka University Teaching Hospital [28], it was found that over 45% of the TB patients could not comply with drug treatemnt instructions for various reasons, including poor transportation to the hospital and lack of family support.

Over 79% of the patients, had suffered from TB only once, while the rest had suffered more than twice. Results present a wide range in the percentage of opposing views for TB as an air borne disease. Within age groups, there was usually no relationship between the documented preventive measures against TB and air borne germs. The reasons were attributed to the fact that these were patients obtaining their drugs from a health facility, hence, received some of the information on disease transmission. The relationship between the awareness 'coughing blood' and 'TB' was explored during the health talks.

## Conclusion

The findings in the Ndola study showed that approximately 39.8% of all TB patients on treatment did not adhere to their treatment schedules, when they started feeling better.

## Competing interests

The author(s) declare that they have no competing interests.

## Authors contributions

FADK was the principal investigator of the study and was responsible for the design, implementation and supervised data entry and cleaning. He worked closely with the biostatistician during data analysis. He is the principal author of this paper. MT was the co-investigator of the study. She contributed to the design of the study, coordinated data collection, entry and cleaning. She was part of the data analysis team. SS is a co-author of this puplication who carried out data analysis as a biostatistician. LS co-authored this publication and was responsible for data entry and cleaning. He was part of the data analysis team. All authors have read and approved the final manuscript.

## Pre-publication history

The pre-publication history for this paper can be accessed here:



## References

[B1] (1997). HIV/AIDS in Zambia: Background Projections Impacts Interventions. Ministry of Health and Central Board of Health Lusaka, Zambia.

[B2] Central Statistical Office [Zambia], Central Board of Health [Zambia], ORC Macro (2003). Zambia Demographic Health Survey *(2001–2002)*. Calverton, Maryland, USA: central Statistical Office, Central Board of Health, and ORC Macro.

[B3] Zambia National HIV/AIDS/TB Council (1999). Zambia National HIV/AIDS Strategic Plan Summary, 1999–2001, Lusaka, Zambia.

[B4] Sukwa TY, Kaona ADF, Musonda MR (1999). Preliminary Report on Multicentre Study on Factors Determining Deferential Spread of HIV in African Towns (Ndola site). Ndola Tropical Diseases Research Centre.

[B5] Horizons Program. Ndola Demonstration Project (2002). a midterm analysis of lessons learned. Nairobi Kenya: Population Council.

[B6] Knut F, Musonda MR, Kasumba K, Ndhovu Z, Mluanda F, Kaetano L, Chipaila CC (1997). The HIV Epidemic in Zambia: Socio-demographic Prevalence Patterns and Indicators of Trends Among Childbearing Women. AIDS.

[B7] Auer C, Sarol JJ, Tanner M, Weiss M (2000). Health seeking and perceived causes of tuberculosis among patients in Manila. J Trop Med Int Health.

[B8] Farmer PE, Robin SL, Kim JY (1991). Tuberculosis, poverty and compliance: lesson from rural Haiti. Sem Respir Infect.

[B9] Farmer PE, Kim JY (1998). Community based approaches to the control of multidrug-resistant tuberculosis. Introducing "DOT" "Plus.". Brit Med J.

[B10] Nyirenda C (1998). Impact of HIV and AIDS on families and children. HIV and Development Programme. Issues Paper # 22E: 10–27 Issued by UNDP and UNICEF in collaboration with UNAIDS.

[B11] Wilkinson D (1994). High compliance tuberculosis treatment Programme in rural community. Lancet.

[B12] Odusanya OO, Babafeni O Joseph (2004). Patterns of delays among pulmonary tuberculosis patients in Lagos, Nigeria. BMC Public Health.

[B13] Ponyk RM, Makhubele MB, Hargreaves JR, Tollman SM, Hausler HP (2001). Assessing health seeking behaviour among tuberculosis patients in South Africa. Int J Tuberc Lung Dis.

[B14] Kaona ADF, Lafort E, Musonda MR, Tembo M, Siajunza TM (2004). Round 1 Behavioral Surveillance Survey Zambia 2000, Volume one and two. Submitted to National AIDS Council-Ministry of Health, Lusaka-Zambia, USAID, Family Health International, Institute of Tropical Medicine, Belgium and World Vision.

[B15] Somerville MA, Orkin AJ (1989). Human rights discrimination and AIDS: Concepts and Issues. AIDS.

[B16] Maher D, Hausler HP, Raviglione MC, Kaleeba N, Aisu T (1997). Fourie- Tuberculosis care in community care organizations in sub-Saharan Africa: practice and potential. Global Tuberculosis Programme, World Health Organization, Geneva, Switzerland. Int J tuberc Lung Dis.

[B17] Needham DM, Fanssett GP, Foster SD (1998). Barriers to tuberculosis Control in urban Zambia: the economic impact and burden on patients prior to diagnosis. Int J Tuberc Lung Dis.

[B18] Steen TW, Mazonde GN, Ngaka Ya Setwana, Ngaka Ya Sekgoa (1999). Health Seeking Behaviour in Botswana with Pulmonary Tuberculosis. Soc Sci Med.

[B19] Ingengo A, Mattossovich D, Kiasekoka MJ, Caprara A, Dedri S, Tap G (1993). AIDS patients in Abjan: Social dynamics and care process. Abstract number (PO-D 20-4014), Berlin.

[B20] Glynn JR, Caraël M, Auvert B, Kahindo M, Chege J, Musonda R, Kaona F, Buvé A, for the Study Group on Heterogeneity of HIV Epidemics in African Cities (2001). "Why do young women have a much higher prevalence of HIV than young men? A study in Kisumu, Kenya and Ndola, Zambia". AIDS.

[B21] Odebiye OI (1992). Conception of AIDS and its prevention in a Nigerian University. J R Soc Hlth.

[B22] Maher D (2002). Strategic framework to decrease the burden of TB/HIV. Stop TB Department.

[B23] Gostin L (1995). Informed consent, cultural sensitivity and respect for persons. J Am Med Ass.

[B24] Kaona FAD, Tuba M, Mwandu D (2002). Ndola Demonstration Project (NDP) – Zambia: Sampling and Recruitment for the Community: Sample for the Evaluation and Impact Assessment of the 18 months follow-up survey. Technical Report of the Mwengu Social and Health Research Center (MSHRC) – Ndola, Zambia.

[B25] Simon D, Adams MA, Madhavan S (2002). Women's Social Power, Child nutrition and Poverty in Mali. J Biosoc Sci.

[B26] Rajeswari R, Chandrasekaran V, Suhadve M, Sivasubramaniam S, Sudha GR (2002). Factors associated with patients and health system delays in the diagnosis of tuberculosis in South India. Int J Tuberc Lung Dis.

[B27] Buvé A, Caraël M, Hayes RJ, Auvert B, Ferry B, Robinson NJ, for the Study Group on Heterogeneity of HIV Epidemics in African Cities (2001). "The multicentre study on factors determining the differential spread of HIV in four African cities: summary and conclusion.". AIDS.

[B28] Luo C, Chintu C, Bhat G, Raviglione M, Diwan V, DuPont HL, Zumla A (1994). Human immunodeficiency virus type-1 infection in Zambian children with tuberculosis: changing seroprevalence and evaluation of a thioacetazone-free regimen. Tuber Lung Dis.

